# Notes and Notices

**Published:** 1885-12

**Authors:** 


### NOTES AND NOTICES.

Supplement for 1886. See advertisement, on page 3.

Dr. C. A. Harmon, of Lancaster, O., proposes prostatotomy for the relief of hypertrophy of prostate gland (vide *St. Louis Periscope* for October).

Dr. Percy O. B. Gause, of Philadelphia, has located for the winter at Aiken, S. C., where he will continue practice. He solicits the care of patients ordered South by their physicians.

Warning.—The *People's Health Journal* of Chicago warns its readers against the abominable habit which many people have of putting money in the mouth. It *is* a dangerous practice, as syphilis may easily be taken in this way.

Dr. William Benjamin Carpenter, LL. D., F. R. S., an eminent English physiologist, has lately died from the effects of terrible burns, caused by the upsetting of a lamp while he was taking a vapor bath for rheumatism.

The Medical News (of November 7th) copies from an exchange an interesting account of a girl born without any vagina. An operation was performed, relieving the uterus of about two quarts of retained menstrual fluid. An artificial vagina having been established, the girl thereafter menstruated regularly.

Sir James Paget has been tracing the course in life of one thousand medical students taken at random from an English institute. He found that twenty-three out of the one thousand achieved distinguished success; sixty-six had considerable success; five hundred and seven made a living; one hundred and twenty-four had a very limited success, not having made a fair practice within fifteen years after graduation, and fifty-six failed utterly. Nearly ten per cent. (ninety-six) of the whole number left the profession after beginning either study or practice, eighty-seven died after entering practice, and forty-one died when students.

"The American Obstetrical Society" is the name of a new association started in New York in October last "to engage in the study of the art and science of obstetrics in a systematic manner, with the hope of making its practice more exact and satisfactory." The officers are: President, George W. Winterburn, M. D., of New York; Vice-Presidents, Henry Minton, M. D., Brooklyn, N. Y.; Professor Sheldon Leavitt, M. D., Chicago; Professor Walter Wesselhœft, M. D., Cambridge, Mass.; Secretary, Everett Hasbrouck, M. D., Brooklyn, N. Y.; Treasurer, Clarence M. Conant, M. D., Orange, N. J. For further particulars apply to the Secretary.

The Bacillus of Cholera.—Those who believe that Asiatic cholera is caused by a specific germ, but are not ready to accept the conclusions of Dr. Robert Koch concerning the comma bacillus and its work, may find support for their theories in a report of a committee of eminent English physicians upon the investigations made by Drs. Klein and Gibbes. * * * * * *

The English investigators found the comma bacilli of Koch, but they do not admit the soundness of his conclusions concerning the power of these organisms. They declare that the bacilli are found only in dead tissues. The committee declares that their report justifies the inference "that no direct relation exists between the number of comma-shaped organisms associated with the choleraic process and the gravity of the disease, and that these organisms are not found in the blood or tissues, and are not ordinarily, if ever, to be found in the tissues of any part of the intestinal canal in even the most acute cases of cholera when the post-mortem examination is made immediately after death." The investigators found the same kind of organisms in cases of intestinal diseases other than cholera. They undertake to show that water containing the comma bacilli has been used with safety in Calcutta by hundreds of families. They declare that it has been proved by their experiments that it is not possible to produce in animals by inoculation with these organisms taken from cholera patients "any illness, be the introduction into the system carried out by feeding, by subcutaneous injection into the jugular vein, or by injection into the cavity of the intestine." They add that there is no direct experimental evidence that cholera can be induced in man by the introduction of a pure cultivation of comma bacilli into his system. * * *
—*The New York Times, November 29th, 1885.*

